# Preface to the theme issue ‘physics-informed machine learning and its structural integrity applications'

**DOI:** 10.1098/rsta.2023.0176

**Published:** 2023-11-13

**Authors:** Shun-Peng Zhu, Abílio M. P. De Jesus, Filippo Berto, John G. Michopoulos, Francesco Iacoviello, Qingyuan Wang

**Affiliations:** ^1^ School of Mechanical and Electrical Engineering, University of Electronic Science and Technology of China, Chengdu 611731, People's Republic of China; ^2^ INEGI, Faculty of Engineering, University of Porto, Porto 4200-465, Portugal; ^3^ Department of Chemical Engineering, Materials and Environment, Sapienza University of Rome, 00184 Roma, Italy; ^4^ Computational Multiphysics Systems Lab, Center for Materials Physics and Technology, Naval Research Laboratory, USA; ^5^ Department of Civil and Mechanical Engineering, University of Cassino and Southern Lazio, Cassino, Italy; ^6^ MOE Key Laboratory of Deep Earth Science and Engineering, College of Architecture and Environment, Sichuan University, Chengdu 610065, People's Republic of China; ^7^ Advanced Research Institute, Chengdu University, Chengdu 610106, People's Republic of China

**Keywords:** machine learning, physics-informed machine learning, structural integrity, failure mechanism modelling, prognostic and health management

## Abstract

The issue focuses on physics-informed machine learning and its applications for structural integrity and safety assessment of engineering systems/facilities. Data science and data mining are fields in fast development with a high potential in several engineering research communities; in particular, advances in machine learning (ML) are undoubtedly enabling significant breakthroughs. However, purely ML models do not necessarily carry physical meaning, nor do they generalize well to scenarios on which they have not been trained on. This is an emerging field of research that potentially will raise a huge impact in the future for designing new materials and structures, and then for their proper final assessment. This issue aims to update the current research state of the art, incorporating physics into ML models, and providing tools when dealing with material science, fatigue and fracture, including new and sophisticated algorithms based on ML techniques to treat data in real-time with high accuracy and productivity.

This article is part of the theme issue 'Physics-informed machine learning and its structural integrity applications (Part 1)'.

Structural integrity is vital for the safe and efficient operation of engineering systems/facilities, including aircraft, wind turbines, power stations, etc. The demands on assessments are constantly increasing as new manufacturing processes and materials are developed and structures are expected to operate within more extreme environments to increase efficiency and reduce environmental impact [1]. With the significantly increased integration of advanced testing and measurement technology into structural health monitoring, structural integrity and safety (SIS) assessment in engineering practice, the assimilation and understanding of the explosive growth and diversity of observational data provide exciting opportunities for the development of high-reliability engineering systems [1–3].

Data science bridges computer science, statistics and domain knowledge to provide a framework which allows for the assimilation of large amounts of observational data into the modelling. Particularly, advances in machine learning (ML) have enabled structural integrity and safety assessment of engineering systems and their improvement through failure mechanism modelling with the combination of either deterministic or probabilistic analyses [4]. Despite some preliminary success in the SIS field, the purely data-driven nature of ML means it is not necessary to obey the fundamental governing laws of physical systems and often fails to describe or predict real scenarios beyond those they have been trained on. Additionally, training deep neural networks normally requires large amounts of quality data, which are not always available for engineering problems.

To solve these challenges, a new paradigm that integrates physical principles into the ML models is emerging: physics-informed machine learning (PIML). Through PIML techniques, studies on structural integrity and performance degradation assessment can be conducted to maximize the lifetime and optimize the inspection and maintenance policy of engineering systems/facilities [5]. Specifically, failure occurs under influences of multi-sources of uncertainty, including load variations in usages, material variability, geometry variations within tolerances and other uncontrolled variations [6].

Incorporating physics into ML models can lead to physically consistent models that are faster to train, and more generalizable, interpretable and trustworthy. Thus, advanced methods and applications for theoretical, numerical and experimental contributions that address these issues on PIML applications in structural integrity and safety assessment of engineering systems/facilities are desired and expected, which attempts to prevent over-design as well as unnecessary inspections, and also provide powerful tools to enable a balance between safety and economy to be achieved.

Accordingly, this theme issue first explores the advances in PIML and its structural integrity applications through accurate failure mechanism modelling combining either deterministic or probabilistic analyses by using computer methods, including artificial intelligence, fuzzy logic, genetic algorithms, intelligent and adaptive systems, ML, neural network computing, etc. Then, this collection discusses several critical issues related to learning from massive amounts of data and highlights current research endeavours and the challenges to data science in structural integrity and safety, especially incorporating physics into ML models, as well as shared recent advances in this research area.

We believe that this theme issue is very timely and will attract interest from both academia and industry. We also hope that the contents of this theme issue will enable more researchers to conduct their research on PIML in their own fields.

## Data Availability

This article has no additional data.
